# Synthesis, crystal structure and Hirshfeld surface analysis of 1-(12-bromo­dodec­yl)indoline-2,3-dione

**DOI:** 10.1107/S2056989023009052

**Published:** 2023-10-19

**Authors:** Nohaila Rharmili, Omar Abdellaoui, Amal Haoudi, Joel T. Mague, Tuncer Hökelek, Fouad Ouazzani Chahdi, Youssef Kandri Rodi, Ahmed Mazzah, Nada Kheira Sebbar

**Affiliations:** a Laboratory of Applied Organic Chemistry, Faculty of Science and Technology, University of Sidi Mohamed Ben Abdellah BP 2202, Fez, Morocco; b Department of Chemistry, Tulane University, New Orleans, LA 70118, USA; c Department of Physics, Hacettepe University, 06800 Beytepe, Ankara, Türkiye; d University of Lille, CNRS, UAR 3290, MSAP, Miniaturization for Synthesis, Analysis and Proteomics, F-59000 Lille, France; e Laboratory of Organic and physical Chemistry, Applied Bioorganic Chemistry Team, Faculty of Sciences, Ibnou Zohr University, Agadir, Morocco; f Laboratory of Heterocyclic Organic Chemistry, Medicines Science Research Center, Pharmacochemistry Competence Center, Mohammed V University in Rabat, Faculty of Sciences, Morocco; University of Aberdeen, United Kingdom

**Keywords:** crystal structure, indoline-2,3-dione, hydrogen bond, dodec­yl, π stacking, inter­calation

## Abstract

In the title compound, C_20_H_28_BrNO_2_, the indoline portion is almost planar and the 12-bromo­dodecyl chain adopts an all-*trans* conformation apart from the *gauche* terminal C—C—C—Br fragment. A micellar-like structure is generated in the crystal by C—H⋯O hydrogen bonds and π-stacking inter­actions between indolinedione head groups and inter­calation of the 12-bromo­dodecyl tails. The Hirshfeld surface analysis of the crystal structure indicates that the most important contributions for the crystal packing are from H⋯H (58.9%), H⋯O/O⋯H (17.9%) and H⋯Br/Br⋯H (9.5%) contacts. A density functional theory (DFT) optimized structure at the B3LYP/ 6–311 G(d,p) level shows good agreement with the experimentally determined mol­ecular structure in the solid state.

## Abstract 2

The pendant dodecyl chain in the title compound adopts an all-*trans* conformation apart from the *gauche* terminal C—C—C—Br moiety.

## 1. Chemical context

The chemistry of isatin (1*H*-indole-2,3-dione; C_8_H_5_NO_2_) and its derivatives has been studied extensively owing to its broad array of uses, particularly within the realms of organic synthesis and medicinal chemistry. The initial reports detailing the synthesis of isatin and its derivatives can be traced back to the early 19th century (Rharmili *et al.*, 2023 ▸; Sonam & Kakkar, 2019 ▸). Nearly two centuries after the publication of these pioneering works, a comprehensive review highlighted the remarkable adaptability of this mol­ecular fragment (Borad *et al.*, 2014 ▸). Isatin derivatives have received much attention due to their properties such as anti-microbial (Pakravan *et al.*, 2013 ▸), anti-mycobacterial (Li *et al.*, 2018 ▸), anti-cancer (Khan *et al.*, 2015 ▸) and corrosion-inhibitory activities (Verma *et al.*, 2023 ▸). As a continuation of our studies in this area (Rharmili *et al.*, 2023 ▸), we now report the synthesis, structure and Hirshfeld surface analysis and DFT computations of the title compound, C_20_H_28_BrNO_2_ (I).

## 2. Structural commentary

**Figure 1:**
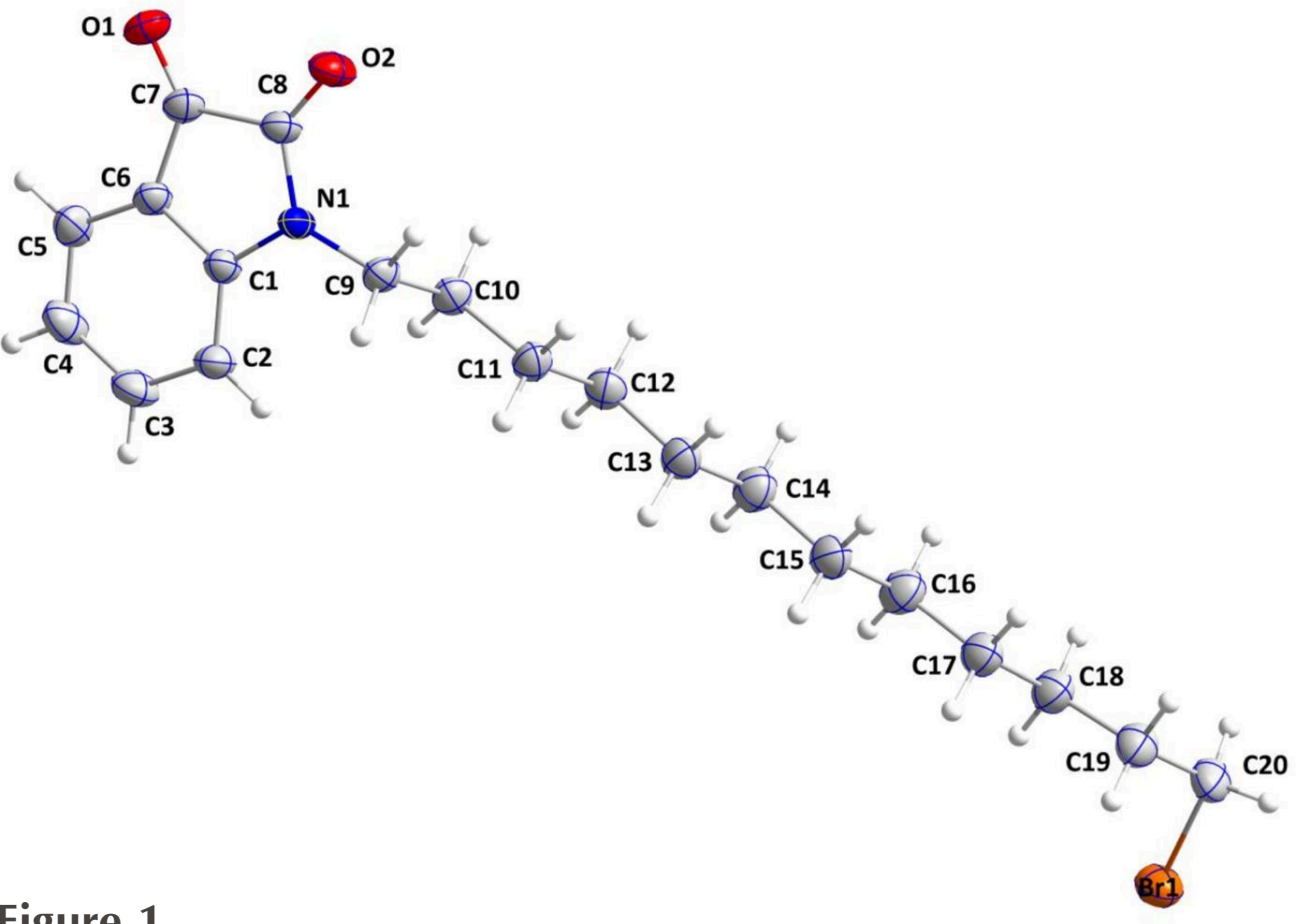
The title mol­ecule showing 50% probability ellipsoids.

As expected, the C1–C8/N1 bicyclic portion of (I) is almost planar (r.m.s. deviation of fitted atoms = 0.007 Å), with C8 showing the largest deviation from the mean plane, by 0.0130 (12) Å. The C10–C20 portion of the dodecyl chain is in an all-*trans* conformation (Fig. 1 ▸), as indicated by the moduli of the torsion angles involving these atoms being within 6° of 180° while the terminal C18—C19—C20—Br1 torsion angle is −70.41 (19)°, indicating a *gauche* conformation. The sum of the bond angles about N1 is 359.9°, suggesting *sp* ^2^ hybridization and involvement of the N lone pair in π bonding with the benzene ring. This is manifested in the C8—N1 bond length of 1.3595 (19) Å as compared with the C1—N1 distance of 1.4113 (19) Å. 
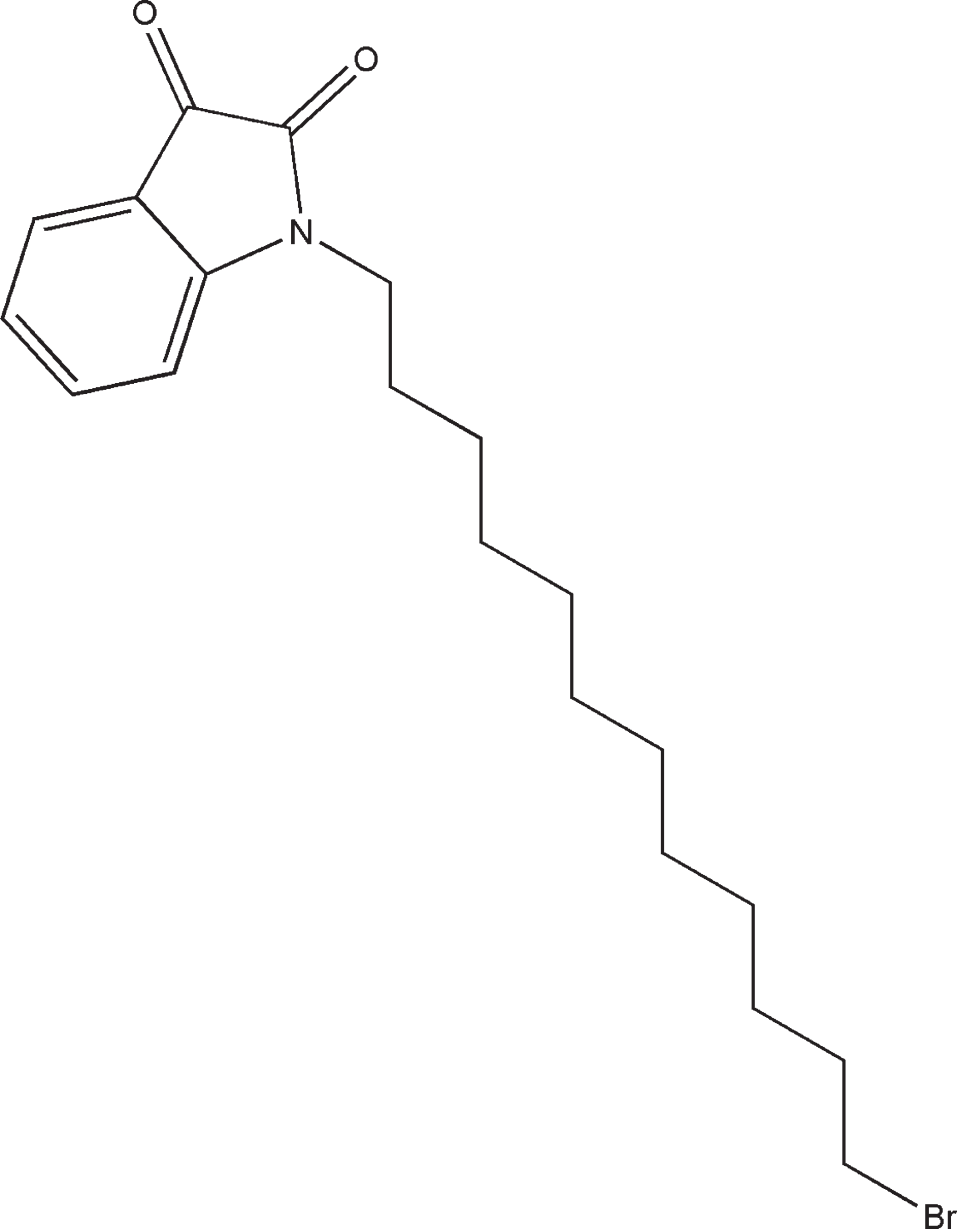


## 3. Supra­molecular features

**Figure 2:**
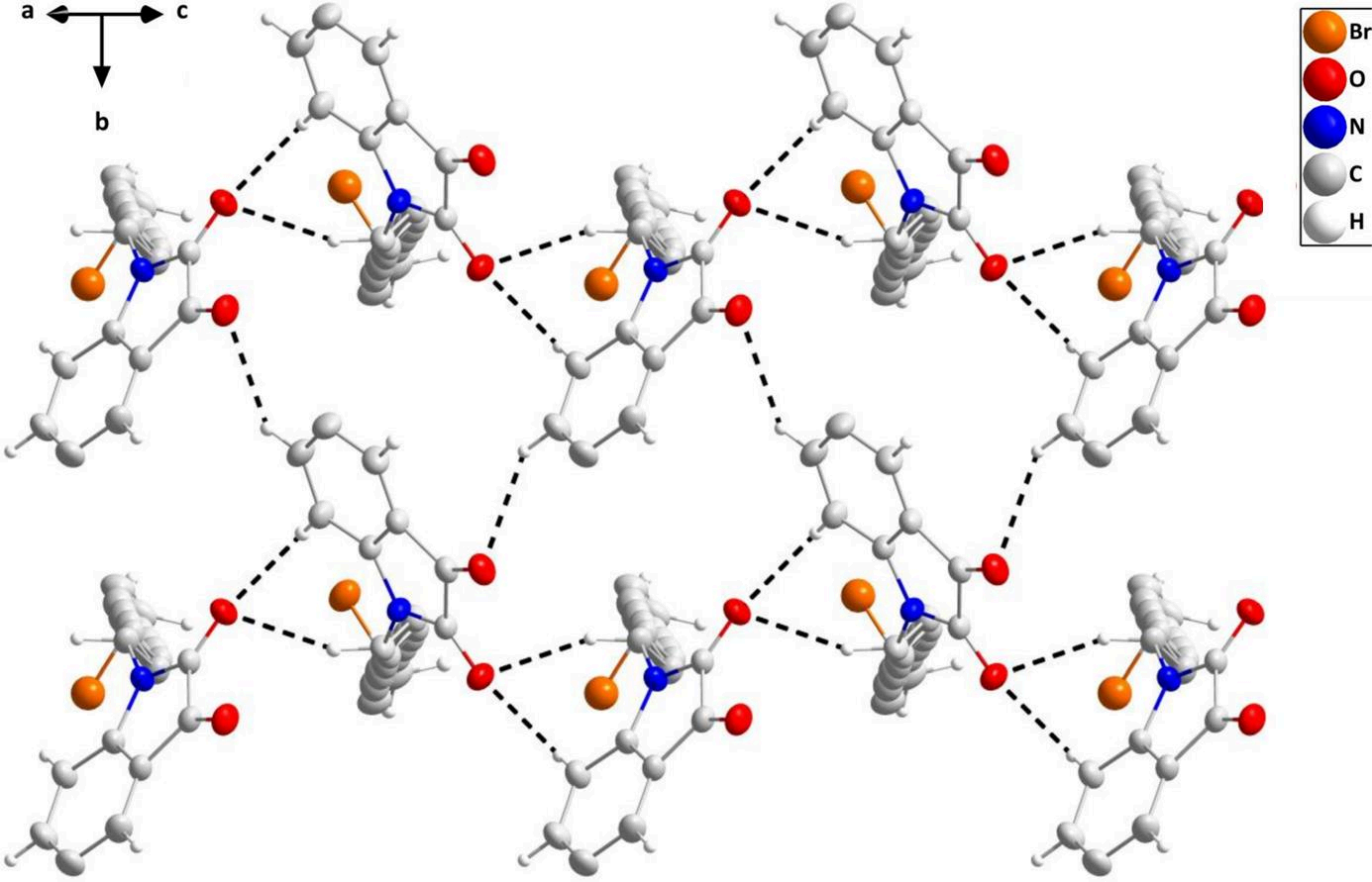
A portion of one layer projected onto (201) with C—H⋯O hydrogen bonds depicted by dashed lines. Non-inter­acting hydrogen atoms are omitted for clarity.

**Figure 3:**
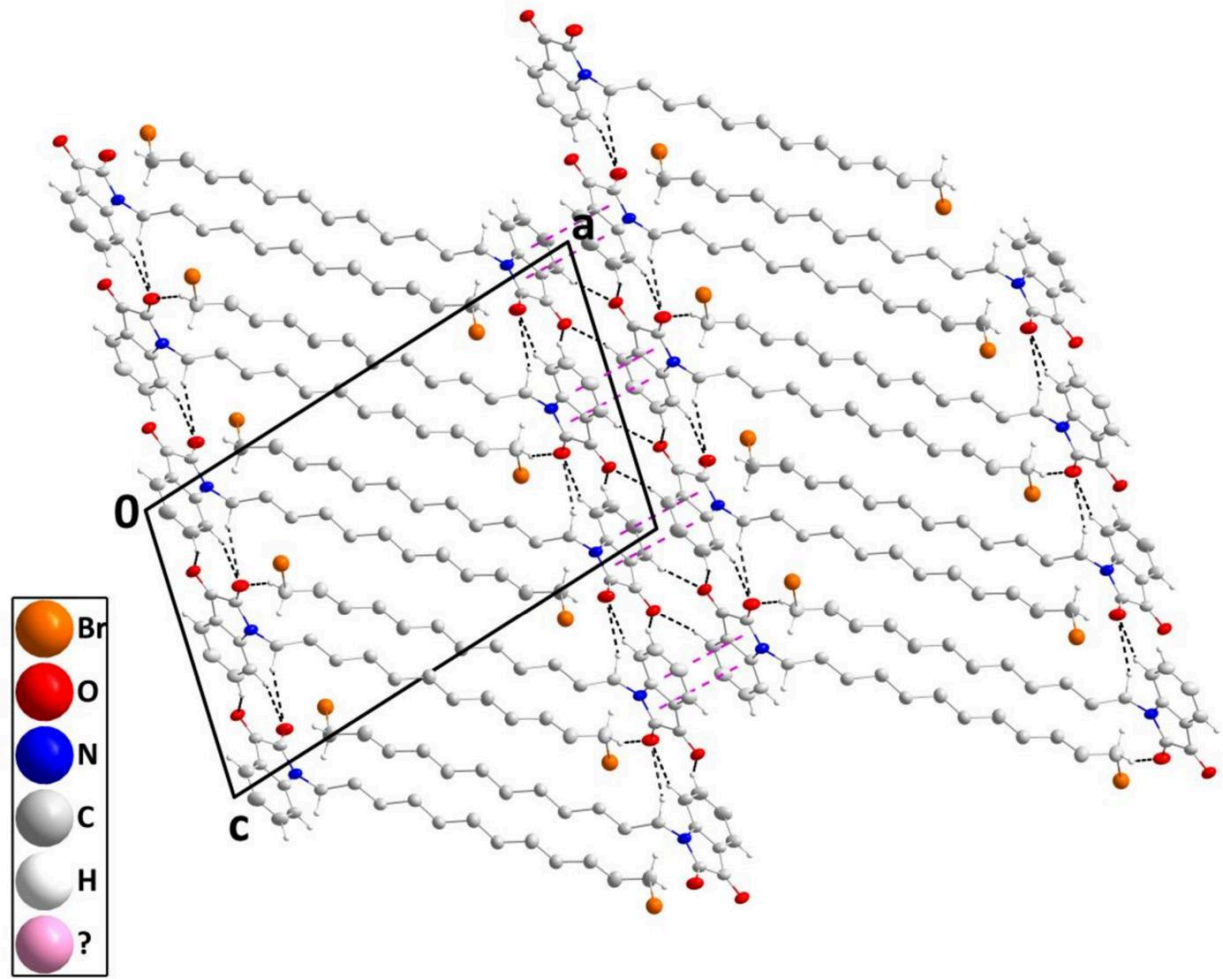
Packing viewed along the *b*-axis direction with C—H⋯O hydrogen bonds and slipped π-stacking inter­actions depicted, respectively, by black and dark pink dashed lines. Non-inter­acting hydrogen atoms are omitted for clarity.

In the crystal, chains of mol­ecules extending along the *c*-axis direction are formed by C2—H2⋯O2 and C9—H9*A*⋯O2 hydrogen bonds (Table 1 ▸) and connected into layers parallel to (201) by C3—H3⋯O1 hydrogen bonds (Table 1 ▸ and Fig. 2 ▸). Pairs of layers are connected head-to-head by C5—H5⋯O1 hydrogen bonds (Table 1 ▸) and slipped π-stacking inter­actions between the five- and six-membered rings [centroid–centroid = 3.6003 (11) Å, dihedral angle = 0.39 (9)°, slippage = 1.35 Å] and these units form a micellar-like structure by inter­calation of the 12-bromo­decyl chains aided by C20—H20*A*⋯O2 hydrogen bonds (Table 1 ▸ and Fig. 3 ▸).

## 4. Hirshfeld surface analysis and DFT calculations

**Figure 4:**
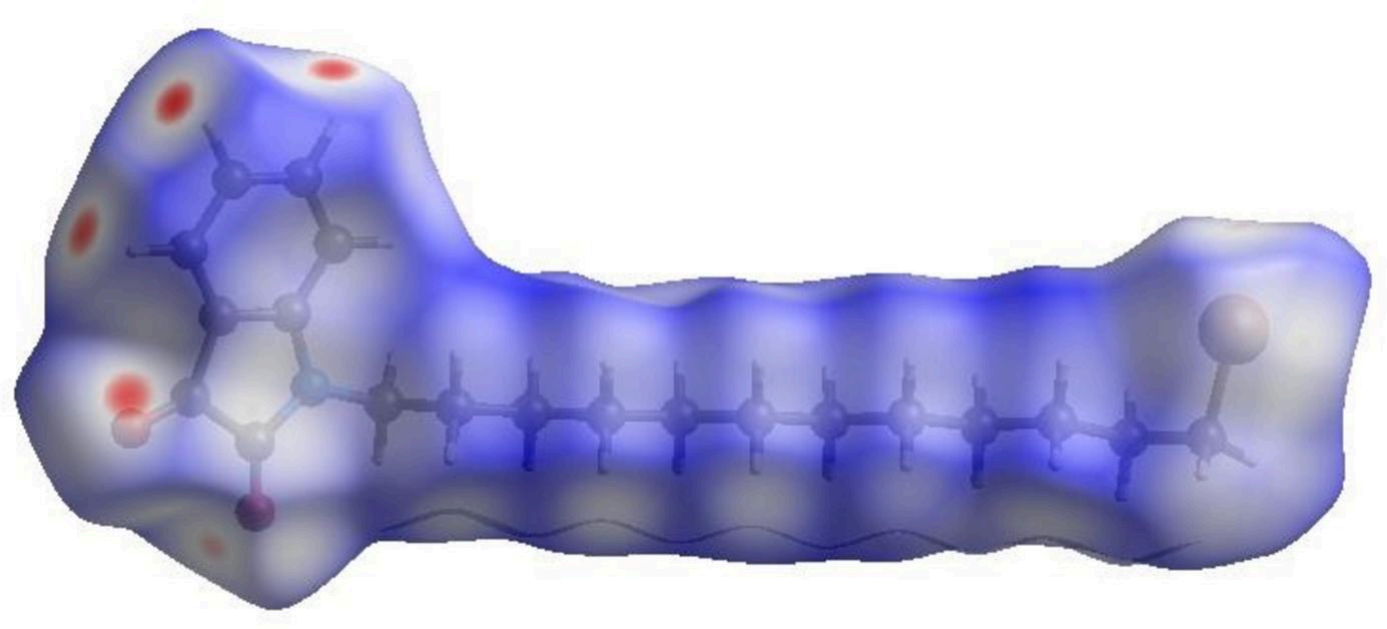
View of the three-dimensional Hirshfeld surface of the title compound, plotted over *d*_norm_ in the range of −0.18 to 1.38 a.u.

**Figure 5:**
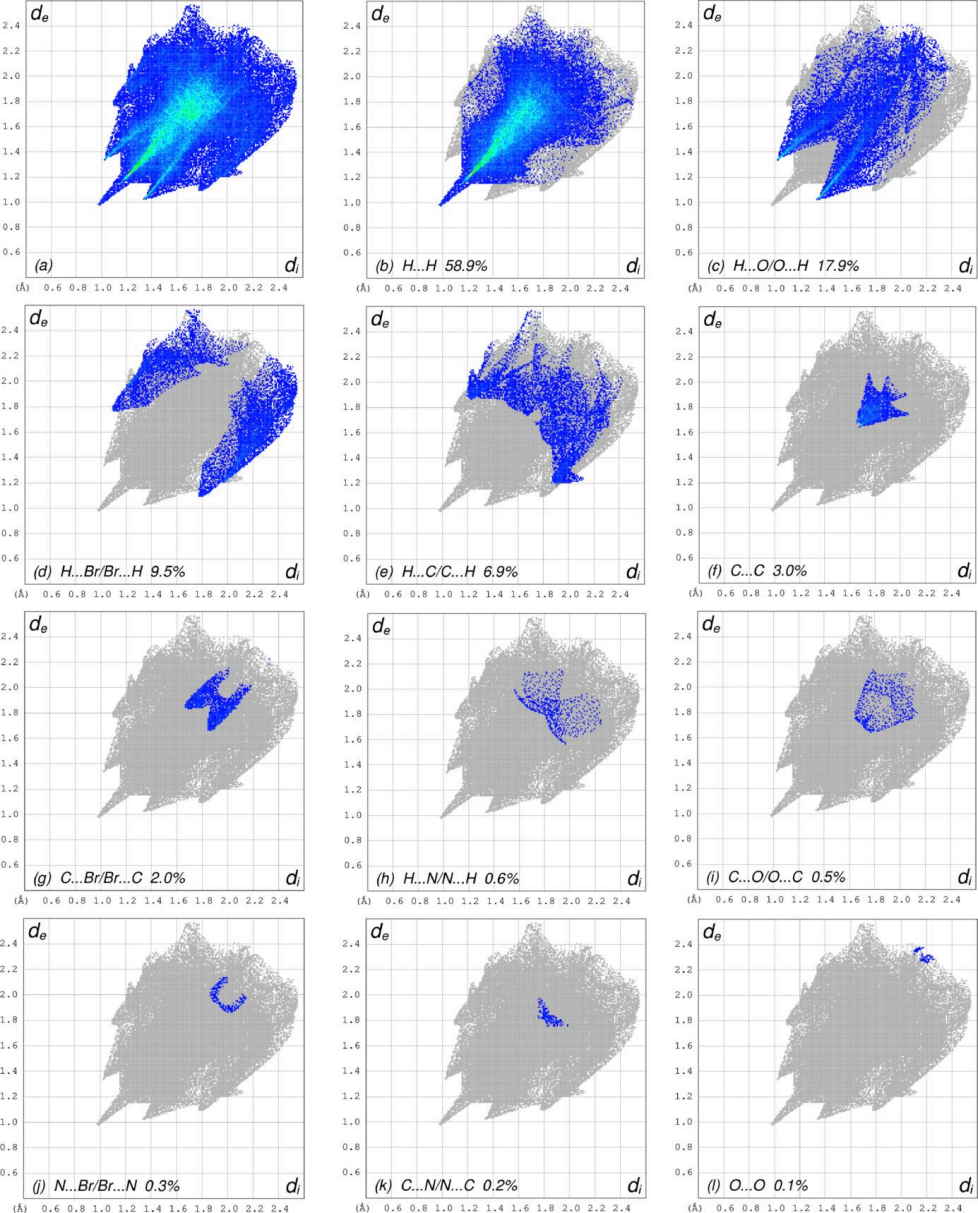
The full two-dimensional fingerprint plots for the title compound, showing (*a*) all inter­actions, (*b*) H⋯H, (*c*) H⋯O/O⋯H, (*d*) H⋯Br/Br⋯H,(*e*) H⋯C/C⋯H, (*f*) C⋯C, (*g*) C⋯Br/Br⋯C, (*h*) H⋯N/N⋯H, (*i*) C⋯O/O⋯C, (*j*) N⋯Br/Br⋯N,, (*k*) C⋯N/N⋯C, (*l*) O⋯O and (*m*) O⋯Br/Br⋯O inter­actions. The *d*_i_ and *d*_e_ values are the closest inter­nal and external distances (in Å) from given points on the Hirshfeld surface contacts.

To further visualize the inter­molecular inter­actions in the crystal of (I), a Hirshfeld surface (HS) analysis was carried out by using *Crystal Explorer 17.5* (Turner *et al.*, 2017 ▸) (Fig. 4 ▸). The red spots indicate their roles as the respective donors and/or acceptors noted above. The overall two-dimensional fingerprint plot, Fig. 5 ▸ *a*, and those delineated into different contact types are illustrated in Fig. 5 ▸ *b*–*m*, respectively, together with their relative contributions to the Hirshfeld surface. The most important inter­action is H⋯H contributing 58.9% to the overall crystal packing, which is reflected in Fig. 5 ▸ *b* as widely scattered points of high density due to the large hydrogen content of the mol­ecule with the tip at *d*_e_ = *d*_i_ = 0.98 Å. The H⋯O/O⋯H contacts contribute 17.9% to the HS, as may be seen in Fig. 5 ▸ *c*, where the symmetric pair of spikes is observed with the tips at *d*_e_ + *d*_i_ = 2.34 Å. The wings of H⋯Br/Br⋯H contacts (Fig. 5 ▸ *d*) are observed with the tips at *d*_e_ + *d*_i_ = 2.88 Å, and a contribution of 9.5% to the HS. In the presence of C—H⋯π inter­actions, the pair of characteristic wings in the fingerprint plot delineated into H⋯C/C⋯H contacts, Fig. 5 ▸ *e*, has a 6.9% contribution to the HS with the tips at *d*_e_ + *d*_i_ = 3.08 Å. The C⋯C contacts (Fig. 5 ▸ *f*), appearing as a bullet-shaped distribution of points, have a contribution of 3.0% to the HS with the tip at *d*_e_ = *d*_i_ = 1.64 Å. The tiny wing pair of C⋯Br/Br⋯C contacts (Fig. 5 ▸ *g*) with a 2.0% contribution to the HS has the tips at *d*_e_ + *d*_i_ = 3.54 Å. Other contact types make a negligible contribution to the HS.

The theoretical structure of (I) was optimized in a gas-phase environment using density functional theory (DFT), using the B3LYP functional and 6-311G(d,p) basis-set calculations (Becke, 1992 ▸), giving an acceptable agreement between observed and calculated geometry (supplementary Table 1): the *R* ^2^ values of the bond lengths and bond angles of (I) were calculated to be 0.998 and 0.991, respectively. The terminal C18—C19—C20—Br1 grouping has observed and calculated torsion angles of −70.41 (19) and 69.06°, respectively. The frontier orbitals of (I) are depicted in supplementary Fig. 1 and the HOMO–LUMO gap of the mol­ecule is about 3.57 eV (supplementary Table 2).

## 5. Database survey

A search conducted in the Cambridge Structural Database (CSD; Version 5.42, last updated in May 2023; Groom *et al.*, 2016 ▸) targeting N-substituted isatin derivatives yielded a total of 58 results. Among these, there were five reports on the structure of isatin itself and four instances of the structure of *N*-methyl­isatin. Thirteen of these structures featured an alkyl chain consisting of two or more carbon atoms. The compound most closely related to the title compound is 1-(3-bromo­prop­yl)-1*H*-indole-2,3-dione (CSD refcode AKOBIN; Qachchachi *et al.*, 2016 ▸), which also features a *gauche* terminal C—C—C—Br grouping.

## 6. Synthesis and crystallization

To a solution of 1*H*-indoline-2,3-dione (2.0 mmol), potassium carbonate (4.0 mmol) and tetra-*n*-butyl­ammonium­bromide (0.20 mmol) in di­methyl­formamide (20 ml) was added 1,12-di­bromo­dodecane (2.2 mmol) and the mixture was then left to stir for 18 h at room temperature. Following salt filtration, the solvent was evaporated at low pressure, and the resulting residue was dissolved in di­chloro­methane. The organic phase was then dried over Na_2_SO_4_ and concentrated. The resulting mixture was chromatographed using a silica gel column with hexa­ne/ethyl­acetate as the eluent (3/1). Single crystals of the title compound suitable for X-ray analysis were obtained by slow evaporation of an ethanol solution.

## 7. Refinement

Crystal data, data collection and structure refinement details are summarized in Table 2 ▸. H atoms attached to carbon were placed in calculated positions (C—H = 0.95–0.99 Å). All were included as riding contributions with isotropic displacement parameters 1.2–1.5 times those of the attached atoms.

## Supplementary Material

Crystal structure: contains datablock(s) global, I. DOI: 10.1107/S2056989023009052/hb8077sup1.cif

Structure factors: contains datablock(s) I. DOI: 10.1107/S2056989023009052/hb8077Isup2.hkl

Supporting information file. DOI: 10.1107/S2056989023009052/hb8077Isup3.cdx

HOMO-LUMO diagram. DOI: 10.1107/S2056989023009052/hb8077sup4.pdf

supplementary Fig. 1. DOI: 10.1107/S2056989023009052/hb8077sup5.png

Supporting information file. DOI: 10.1107/S2056989023009052/hb8077Isup6.cml

CCDC reference: 2301451

Additional supporting information:  crystallographic information; 3D view; checkCIF report

## Figures and Tables

**Table 1:** Hydrogen-bond geometry (Å, °) *Cg*2 is the centroid of the C1–C6 benzene ring.

*D*—H⋯*A*	*D*—H	H⋯*A*	*D*⋯*A*	*D*—H⋯*A*
C2—H2⋯O2^i^	0.95	2.56	3.441 (2)	154
C3—H3⋯O1^ii^	0.95	2.51	3.271 (2)	137
C5—H5⋯O1^iii^	0.95	2.51	3.424 (2)	160
C9—H9*A*⋯O2^i^	0.99	2.60	3.503 (2)	152
C20—H20*A*⋯O2^iv^	0.99	2.47	3.393 (2)	156
C20—H20*B*⋯*Cg*2^v^	0.99	2.96	3.756 (2)	139

Symmetry codes: (i) 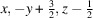 ; (ii) 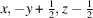 ; (iii) 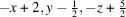 ; (iv) 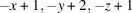 ; (v) 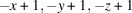 .

**Table 2:** Experimental details

Crystal data	
Chemical formula	C_20_H_28_BrNO_2_
*M*_r_	394.34
Crystal system, space group	Monoclinic, *P*2_1_/*c*
Temperature (K)	150
*a*, *b*, *c* (Å)	20.5385 (5), 8.1977 (2), 12.3185 (3)
β (°)	105.231 (1)
*V* (Å^3^)	2001.19 (8)
*Z*	4
Radiation type	Cu *K*α
μ (mm^−1^)	2.88
Crystal size (mm)	0.23 × 0.07 × 0.05

Data collection	
Diffractometer	Bruker D8 VENTURE PHOTON 3 CPAD
Absorption correction	Multi-scan (*SADABS*; Krause *et al.*, 2015 ▸)
*T*_min_, *T*_max_	0.74, 0.87
No. of measured, independent and observed [*I* > 2σ(*I*)] reflections	42466, 4091, 3810
*R*_int_	0.038
(sin θ/λ)_max_ (Å^−1^)	0.626

Refinement	
*R*[*F* ^2^ > 2σ(*F* ^2^)], *wR*(*F* ^2^), *S*	0.030, 0.084, 1.06
No. of reflections	4091
No. of parameters	217
H-atom treatment	H-atom parameters constrained
Δρ_max_, Δρ_min_ (e Å^−3^)	0.44, −0.66

Computer programs: *APEX4* and *SAINT* (Bruker, 2021 ▸), *SHELXT* (Sheldrick, 2015*a* ▸), *SHELXL2019/1* (Sheldrick, 2015*b* ▸), *DIAMOND* (Brandenburg & Putz, 2012 ▸) and *SHELXTL* (Sheldrick, 2008 ▸).

## supplementary crystallographic information

### Crystal data

**Table:**

C_20_H_28_BrNO_2_	*F*(000) = 824
*M**_r_* = 394.34	*D*_x_ = 1.309 Mg m^−^^3^
Monoclinic, *P*2_1_/*c*	Cu *K*α radiation, λ = 1.54178 Å
*a* = 20.5385 (5) Å	Cell parameters from 9620 reflections
*b* = 8.1977 (2) Å	θ = 6.6–74.7°
*c* = 12.3185 (3) Å	µ = 2.88 mm^−^^1^
β = 105.231 (1)°	*T* = 150 K
*V* = 2001.19 (8) Å^3^	Column, yellow
*Z* = 4	0.23 × 0.07 × 0.05 mm

### Data collection

**Table:**

Bruker D8 VENTURE PHOTON 3 CPAD diffractometer	4091 independent reflections
Radiation source: INCOATEC IµS micro—-focus source	3810 reflections with *I* > 2σ(*I*)
Mirror monochromator	*R*_int_ = 0.038
Detector resolution: 7.3910 pixels mm^-1^	θ_max_ = 74.7°, θ_min_ = 5.8°
φ and ω scans	*h* = −25→25
Absorption correction: multi-scan (*SADABS*; Krause *et al.*, 2015)	*k* = −10→10
*T*_min_ = 0.74, *T*_max_ = 0.87	*l* = −15→15
42466 measured reflections	

### Refinement

**Table:**

Refinement on *F*^2^	Primary atom site location: dual
Least-squares matrix: full	Secondary atom site location: difference Fourier map
*R*[*F*^2^ > 2σ(*F*^2^)] = 0.030	Hydrogen site location: inferred from neighbouring sites
*wR*(*F*^2^) = 0.084	H-atom parameters constrained
*S* = 1.06	*w* = 1/[σ^2^(*F*_o_^2^) + (0.0459*P*)^2^ + 0.8387*P*] where *P* = (*F*_o_^2^ + 2*F*_c_^2^)/3
4091 reflections	(Δ/σ)_max_ = 0.001
217 parameters	Δρ_max_ = 0.44 e Å^−^^3^
0 restraints	Δρ_min_ = −0.66 e Å^−^^3^

### Special details

**Table:**

Experimental. The diffraction data were obtained from 14 sets of frames, each of width 0.5° in ω or φ, collected with scan parameters determined by the "strategy" routine in *APEX4*. The scan time was θ-dependent and ranged from 3 to 12 sec/frame.
Geometry. All esds (except the esd in the dihedral angle between two l.s. planes) are estimated using the full covariance matrix. The cell esds are taken into account individually in the estimation of esds in distances, angles and torsion angles; correlations between esds in cell parameters are only used when they are defined by crystal symmetry. An approximate (isotropic) treatment of cell esds is used for estimating esds involving l.s. planes.
Refinement. Refinement of F^2^ against ALL reflections. The weighted R-factor wR and goodness of fit S are based on F^2^, conventional R-factors R are based on F, with F set to zero for negative F^2^. The threshold expression of F^2^ > 2sigma(F^2^) is used only for calculating R-factors(gt) etc. and is not relevant to the choice of reflections for refinement. R-factors based on F^2^ are statistically about twice as large as those based on F, and R- factors based on ALL data will be even larger. H-atoms attached to carbon were placed in calculated positions (C—H = 0.95 - 0.99 Å). All were included as riding contributions with isotropic displacement parameters 1.2 - 1.5 times those of the attached atoms.

### Fractional atomic coordinates and isotropic or equivalent isotropic displacement parameters (Å^2^)

**Table:**

	*x*	*y*	*z*	*U*_iso_*/*U*_eq_	
Br1	0.23573 (2)	0.63104 (3)	−0.09416 (2)	0.04517 (9)	
O1	0.94289 (6)	0.56654 (16)	1.23349 (9)	0.0368 (3)	
O2	0.85863 (7)	0.82935 (15)	1.10434 (10)	0.0389 (3)	
N1	0.86491 (7)	0.66570 (16)	0.95494 (10)	0.0284 (3)	
C1	0.89366 (8)	0.51288 (19)	0.94215 (12)	0.0279 (3)	
C2	0.89087 (9)	0.4326 (2)	0.84263 (13)	0.0342 (3)	
H2	0.868176	0.478183	0.771913	0.041*	
C3	0.92292 (10)	0.2814 (2)	0.85063 (15)	0.0408 (4)	
H3	0.921145	0.221844	0.783763	0.049*	
C4	0.95722 (11)	0.2157 (2)	0.95316 (17)	0.0441 (4)	
H4	0.978553	0.112444	0.955471	0.053*	
C5	0.96083 (9)	0.2996 (2)	1.05346 (15)	0.0381 (4)	
H5	0.984786	0.255493	1.123970	0.046*	
C6	0.92842 (8)	0.44880 (19)	1.04685 (12)	0.0291 (3)	
C7	0.92153 (8)	0.5672 (2)	1.13243 (12)	0.0295 (3)	
C8	0.87763 (8)	0.70783 (19)	1.06527 (12)	0.0288 (3)	
C9	0.82336 (8)	0.75912 (19)	0.86123 (13)	0.0305 (3)	
H9A	0.846127	0.762987	0.799706	0.037*	
H9B	0.819151	0.872468	0.886282	0.037*	
C10	0.75317 (8)	0.6868 (2)	0.81630 (13)	0.0331 (3)	
H10A	0.727660	0.697533	0.873860	0.040*	
H10B	0.756986	0.569283	0.800587	0.040*	
C11	0.71523 (8)	0.7737 (2)	0.70892 (13)	0.0341 (3)	
H11A	0.711684	0.890940	0.725920	0.041*	
H11B	0.741989	0.765068	0.653024	0.041*	
C12	0.64480 (9)	0.7080 (2)	0.65650 (15)	0.0368 (4)	
H12A	0.616860	0.722365	0.710190	0.044*	
H12B	0.647686	0.589718	0.642263	0.044*	
C13	0.61065 (9)	0.7937 (2)	0.54628 (15)	0.0383 (4)	
H13A	0.607081	0.911480	0.561449	0.046*	
H13B	0.639655	0.782183	0.494000	0.046*	
C14	0.54078 (9)	0.7290 (2)	0.48892 (16)	0.0417 (4)	
H14A	0.511876	0.738333	0.541513	0.050*	
H14B	0.544331	0.611842	0.471812	0.050*	
C15	0.50706 (9)	0.8189 (3)	0.38039 (15)	0.0409 (4)	
H15A	0.502037	0.935261	0.398112	0.049*	
H15B	0.536913	0.813268	0.329128	0.049*	
C16	0.43816 (10)	0.7513 (3)	0.31976 (16)	0.0441 (4)	
H16A	0.409126	0.750231	0.372436	0.053*	
H16B	0.443516	0.637118	0.297345	0.053*	
C17	0.40299 (9)	0.8494 (2)	0.21510 (16)	0.0407 (4)	
H17A	0.396679	0.962919	0.237824	0.049*	
H17B	0.432541	0.852711	0.163291	0.049*	
C18	0.33462 (9)	0.7797 (2)	0.15255 (15)	0.0401 (4)	
H18A	0.341349	0.670770	0.122508	0.048*	
H18B	0.306601	0.765498	0.206143	0.048*	
C19	0.29721 (10)	0.8881 (2)	0.05611 (17)	0.0414 (4)	
H19A	0.292143	0.998012	0.086216	0.050*	
H19B	0.324937	0.899538	0.001841	0.050*	
C20	0.22811 (10)	0.8256 (2)	−0.00592 (17)	0.0439 (4)	
H20A	0.203421	0.911798	−0.056606	0.053*	
H20B	0.202084	0.798731	0.048907	0.053*	

### Atomic displacement parameters (Å^2^)

**Table:**

	*U*^11^	*U*^22^	*U*^33^	*U*^12^	*U*^13^	*U*^23^
Br1	0.04657 (13)	0.04566 (14)	0.04404 (13)	−0.00114 (8)	0.01324 (9)	−0.01268 (8)
O1	0.0439 (6)	0.0442 (7)	0.0219 (5)	−0.0049 (5)	0.0078 (4)	0.0027 (5)
O2	0.0566 (7)	0.0320 (6)	0.0324 (6)	0.0056 (5)	0.0192 (5)	−0.0036 (5)
N1	0.0378 (7)	0.0268 (6)	0.0222 (6)	0.0072 (5)	0.0106 (5)	0.0020 (5)
C1	0.0337 (7)	0.0273 (7)	0.0249 (7)	0.0043 (6)	0.0116 (6)	0.0020 (6)
C2	0.0437 (9)	0.0350 (8)	0.0255 (7)	0.0052 (7)	0.0120 (6)	−0.0016 (6)
C3	0.0544 (10)	0.0344 (9)	0.0381 (9)	0.0054 (8)	0.0202 (8)	−0.0067 (7)
C4	0.0571 (11)	0.0319 (9)	0.0494 (10)	0.0138 (8)	0.0246 (9)	0.0036 (7)
C5	0.0446 (9)	0.0365 (9)	0.0360 (8)	0.0110 (7)	0.0153 (7)	0.0110 (7)
C6	0.0351 (7)	0.0303 (8)	0.0239 (7)	0.0034 (6)	0.0114 (6)	0.0041 (6)
C7	0.0346 (7)	0.0314 (8)	0.0243 (7)	−0.0012 (6)	0.0109 (6)	0.0033 (6)
C8	0.0374 (8)	0.0285 (7)	0.0229 (7)	0.0006 (6)	0.0124 (6)	0.0004 (6)
C9	0.0374 (8)	0.0297 (7)	0.0255 (7)	0.0056 (6)	0.0103 (6)	0.0067 (6)
C10	0.0368 (8)	0.0338 (8)	0.0302 (7)	0.0029 (6)	0.0113 (6)	0.0063 (6)
C11	0.0339 (8)	0.0383 (8)	0.0306 (8)	0.0028 (6)	0.0096 (6)	0.0071 (6)
C12	0.0353 (8)	0.0396 (9)	0.0365 (8)	0.0003 (7)	0.0114 (7)	0.0037 (7)
C13	0.0327 (8)	0.0468 (10)	0.0357 (8)	0.0007 (7)	0.0098 (7)	0.0031 (7)
C14	0.0365 (9)	0.0459 (10)	0.0413 (9)	−0.0022 (7)	0.0079 (7)	0.0006 (8)
C15	0.0333 (8)	0.0515 (10)	0.0379 (9)	−0.0005 (8)	0.0093 (7)	0.0002 (8)
C16	0.0389 (9)	0.0501 (10)	0.0401 (9)	−0.0043 (8)	0.0050 (7)	−0.0003 (8)
C17	0.0367 (9)	0.0468 (10)	0.0377 (9)	−0.0005 (7)	0.0084 (7)	−0.0011 (7)
C18	0.0384 (9)	0.0433 (9)	0.0367 (9)	−0.0009 (7)	0.0067 (7)	−0.0011 (7)
C19	0.0449 (10)	0.0335 (8)	0.0429 (9)	0.0040 (7)	0.0062 (8)	−0.0043 (7)
C20	0.0440 (10)	0.0399 (9)	0.0442 (10)	0.0116 (8)	0.0050 (8)	−0.0084 (8)

### Geometric parameters (Å, º)

**Table:**

Br1—C20	1.9593 (18)	C12—C13	1.526 (2)
O1—C7	1.2064 (19)	C12—H12A	0.9900
O2—C8	1.214 (2)	C12—H12B	0.9900
N1—C8	1.3595 (19)	C13—C14	1.519 (2)
N1—C1	1.4113 (19)	C13—H13A	0.9900
N1—C9	1.4593 (19)	C13—H13B	0.9900
C1—C2	1.379 (2)	C14—C15	1.524 (3)
C1—C6	1.402 (2)	C14—H14A	0.9900
C2—C3	1.395 (2)	C14—H14B	0.9900
C2—H2	0.9500	C15—C16	1.521 (3)
C3—C4	1.384 (3)	C15—H15A	0.9900
C3—H3	0.9500	C15—H15B	0.9900
C4—C5	1.399 (3)	C16—C17	1.530 (3)
C4—H4	0.9500	C16—H16A	0.9900
C5—C6	1.385 (2)	C16—H16B	0.9900
C5—H5	0.9500	C17—C18	1.524 (2)
C6—C7	1.467 (2)	C17—H17A	0.9900
C7—C8	1.560 (2)	C17—H17B	0.9900
C9—C10	1.522 (2)	C18—C19	1.521 (3)
C9—H9A	0.9900	C18—H18A	0.9900
C9—H9B	0.9900	C18—H18B	0.9900
C10—C11	1.524 (2)	C19—C20	1.514 (3)
C10—H10A	0.9900	C19—H19A	0.9900
C10—H10B	0.9900	C19—H19B	0.9900
C11—C12	1.519 (2)	C20—H20A	0.9900
C11—H11A	0.9900	C20—H20B	0.9900
C11—H11B	0.9900		
			
C8—N1—C1	111.16 (12)	H12A—C12—H12B	107.9
C8—N1—C9	125.20 (13)	C14—C13—C12	114.24 (15)
C1—N1—C9	123.53 (13)	C14—C13—H13A	108.7
C2—C1—C6	122.08 (14)	C12—C13—H13A	108.7
C2—C1—N1	127.01 (14)	C14—C13—H13B	108.7
C6—C1—N1	110.90 (13)	C12—C13—H13B	108.7
C1—C2—C3	116.91 (15)	H13A—C13—H13B	107.6
C1—C2—H2	121.5	C13—C14—C15	113.28 (16)
C3—C2—H2	121.5	C13—C14—H14A	108.9
C4—C3—C2	121.89 (16)	C15—C14—H14A	108.9
C4—C3—H3	119.1	C13—C14—H14B	108.9
C2—C3—H3	119.1	C15—C14—H14B	108.9
C3—C4—C5	120.73 (16)	H14A—C14—H14B	107.7
C3—C4—H4	119.6	C16—C15—C14	113.84 (16)
C5—C4—H4	119.6	C16—C15—H15A	108.8
C6—C5—C4	117.98 (16)	C14—C15—H15A	108.8
C6—C5—H5	121.0	C16—C15—H15B	108.8
C4—C5—H5	121.0	C14—C15—H15B	108.8
C5—C6—C1	120.39 (14)	H15A—C15—H15B	107.7
C5—C6—C7	132.69 (15)	C15—C16—C17	113.24 (16)
C1—C6—C7	106.93 (13)	C15—C16—H16A	108.9
O1—C7—C6	131.37 (15)	C17—C16—H16A	108.9
O1—C7—C8	123.61 (15)	C15—C16—H16B	108.9
C6—C7—C8	105.03 (12)	C17—C16—H16B	108.9
O2—C8—N1	127.38 (15)	H16A—C16—H16B	107.7
O2—C8—C7	126.65 (14)	C18—C17—C16	113.39 (16)
N1—C8—C7	105.96 (12)	C18—C17—H17A	108.9
N1—C9—C10	112.60 (13)	C16—C17—H17A	108.9
N1—C9—H9A	109.1	C18—C17—H17B	108.9
C10—C9—H9A	109.1	C16—C17—H17B	108.9
N1—C9—H9B	109.1	H17A—C17—H17B	107.7
C10—C9—H9B	109.1	C19—C18—C17	112.64 (16)
H9A—C9—H9B	107.8	C19—C18—H18A	109.1
C9—C10—C11	110.67 (13)	C17—C18—H18A	109.1
C9—C10—H10A	109.5	C19—C18—H18B	109.1
C11—C10—H10A	109.5	C17—C18—H18B	109.1
C9—C10—H10B	109.5	H18A—C18—H18B	107.8
C11—C10—H10B	109.5	C20—C19—C18	114.25 (16)
H10A—C10—H10B	108.1	C20—C19—H19A	108.7
C12—C11—C10	114.56 (14)	C18—C19—H19A	108.7
C12—C11—H11A	108.6	C20—C19—H19B	108.7
C10—C11—H11A	108.6	C18—C19—H19B	108.7
C12—C11—H11B	108.6	H19A—C19—H19B	107.6
C10—C11—H11B	108.6	C19—C20—Br1	110.79 (13)
H11A—C11—H11B	107.6	C19—C20—H20A	109.5
C11—C12—C13	112.25 (14)	Br1—C20—H20A	109.5
C11—C12—H12A	109.2	C19—C20—H20B	109.5
C13—C12—H12A	109.2	Br1—C20—H20B	109.5
C11—C12—H12B	109.2	H20A—C20—H20B	108.1
C13—C12—H12B	109.2		
			
C8—N1—C1—C2	−179.95 (16)	C9—N1—C8—O2	−1.9 (3)
C9—N1—C1—C2	3.8 (3)	C1—N1—C8—C7	1.52 (17)
C8—N1—C1—C6	−1.14 (19)	C9—N1—C8—C7	177.72 (14)
C9—N1—C1—C6	−177.41 (14)	O1—C7—C8—O2	−1.5 (3)
C6—C1—C2—C3	1.6 (3)	C6—C7—C8—O2	178.29 (16)
N1—C1—C2—C3	−179.68 (16)	O1—C7—C8—N1	178.80 (15)
C1—C2—C3—C4	−1.3 (3)	C6—C7—C8—N1	−1.37 (16)
C2—C3—C4—C5	0.1 (3)	C8—N1—C9—C10	−101.92 (18)
C3—C4—C5—C6	0.8 (3)	C1—N1—C9—C10	73.83 (19)
C4—C5—C6—C1	−0.5 (3)	N1—C9—C10—C11	−172.10 (13)
C4—C5—C6—C7	179.77 (18)	C9—C10—C11—C12	179.03 (14)
C2—C1—C6—C5	−0.8 (3)	C10—C11—C12—C13	−177.09 (15)
N1—C1—C6—C5	−179.66 (15)	C11—C12—C13—C14	178.41 (15)
C2—C1—C6—C7	179.05 (15)	C12—C13—C14—C15	178.72 (16)
N1—C1—C6—C7	0.16 (18)	C13—C14—C15—C16	177.83 (17)
C5—C6—C7—O1	0.3 (3)	C14—C15—C16—C17	176.30 (16)
C1—C6—C7—O1	−179.48 (17)	C15—C16—C17—C18	178.65 (16)
C5—C6—C7—C8	−179.49 (18)	C16—C17—C18—C19	174.02 (16)
C1—C6—C7—C8	0.71 (16)	C17—C18—C19—C20	−178.23 (16)
C1—N1—C8—O2	−178.14 (16)	C18—C19—C20—Br1	−70.41 (19)

### Hydrogen-bond geometry (Å, º)

*Cg*2 is the centroid of the C1–C6 benzene ring.

**Table:**

*D*—H···*A*	*D*—H	H···*A*	*D*···*A*	*D*—H···*A*
C2—H2···O2^i^	0.95	2.56	3.441 (2)	154
C3—H3···O1^ii^	0.95	2.51	3.271 (2)	137
C5—H5···O1^iii^	0.95	2.51	3.424 (2)	160
C9—H9*A*···O2^i^	0.99	2.60	3.503 (2)	152
C20—H20*A*···O2^iv^	0.99	2.47	3.393 (2)	156
C20—H20*B*···*Cg*2^v^	0.99	2.96	3.756 (2)	139

Symmetry codes: (i) *x*, −*y*+3/2, *z*−1/2; (ii) *x*, −*y*+1/2, *z*−1/2; (iii) −*x*+2, *y*−1/2, −*z*+5/2; (iv) −*x*+1, −*y*+2, −*z*+1; (v) −*x*+1, −*y*+1, −*z*+1.

### Comparison of the selected (X-ray and DFT) geometric data (Å, °)

**Table:**

Bonds/angles	X-ray	B3LYP/6-311G(d,p)
Br1-C20	1.9593 (18)	2.001
O1-C7	1.2064 (19)	1.2341
O2-C8	1.214 (2)	1.236
N1-C8	1.3595 (19)	1.391
N1-C1	1.4113 (19)	1.417
N1-C9	1.4593 (19)	1.465
C8-N1-C1	111.16 (12)	110.94
C8-N1-C9	125.20 (13)	124.87
C1-N1-C9	123.53 (13)	122.98
C2-C1-C6	122.08 (14)	122.69
C2-C1-N1	127.01 (14)	128.00
C19-C20-Br1	110.79 (13)	110.97

### Calculated energies.

**Table:**

Molecular Energy (a.u.) (eV)	Compound (I)
Total Energy *TE* (eV)	-96820.71
E_HOMO_ (eV)	-6.62
E_LUMO_ (eV)	-3.05
Gap *ΔE* (eV)	3.57
Dipole moment *µ* (Debye)	5.14
Ionisation potential *I* (eV)	6.62
Electron affinity *A*	3.05
Electronegativity *χ*	-4.83
Hardness *η*	-1.78
Softness *σ*	-0.56
Electrophilicity index *ω*	-6.53
