# Supplementary figures and images for: Synthesis, crystal structure and Hirshfeld surface analysis of 1-(12-bromo­dodec­yl)indoline-2,3-dione
Source: Acta Crystallogr E Crystallogr Commun. 2023 Oct 19;79(Pt 11):1033–6. doi: 10.1107/S2056989023009052 (PMC10626969; doi:10.1107/S2056989023009052)

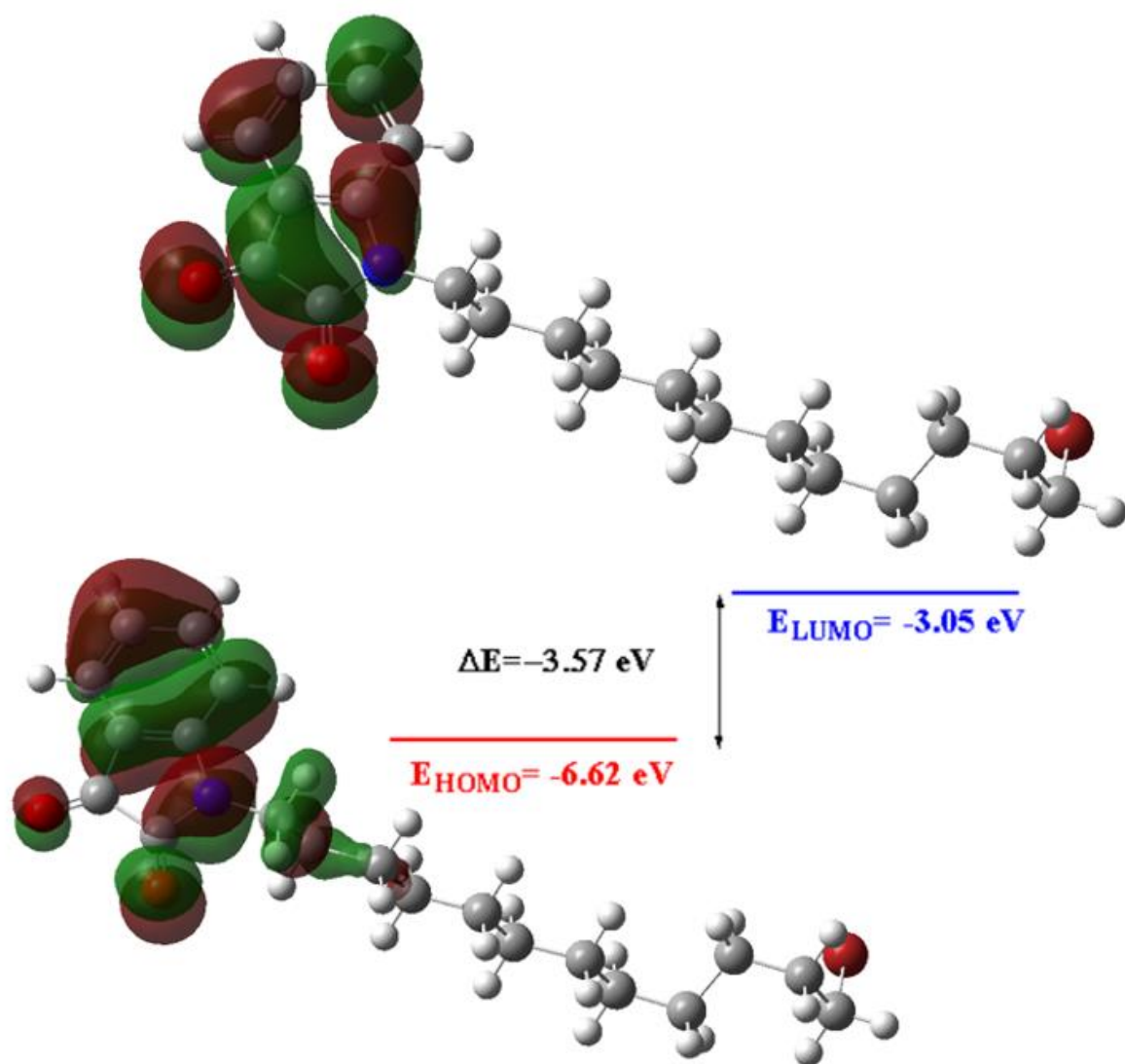

Supplement: Supplementary file 4 [file e-79-01033-sup4.pdf]

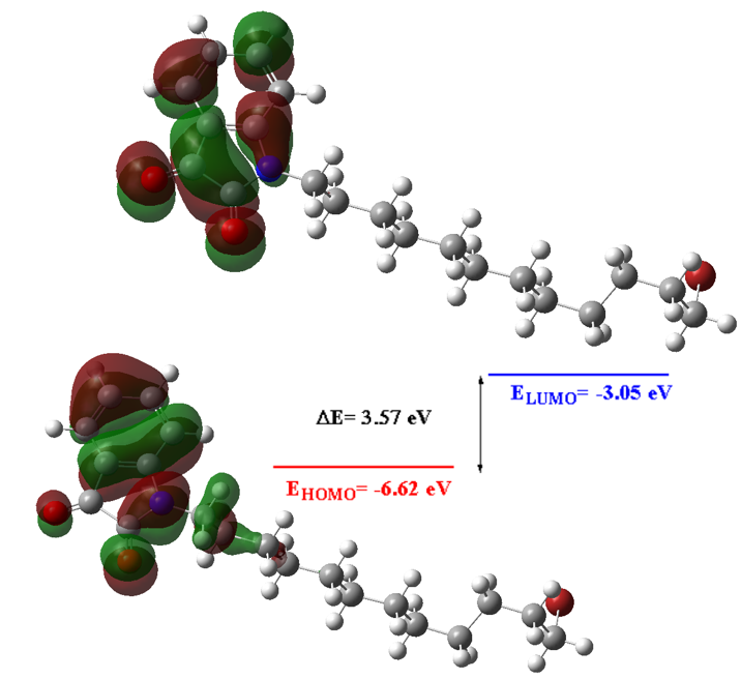

Supplement: Supplementary file 5 [file e-79-01033-sup5.png]
